# Limb‐shaking transient ischemic attack with facial muscles involuntary twitch successfully treated with internal carotid artery stenting

**DOI:** 10.1002/brb3.1679

**Published:** 2020-05-30

**Authors:** Yuan‐yuan Han, Dong Qi, Xiao‐Dong Chen, Chun‐Jie Song

**Affiliations:** ^1^ Department of Neurology Suqian First Hospital Suqian China

**Keywords:** angioplasty, carotid stenosis, internal carotid artery, limb‐shaking transient ischemic attack

## Abstract

**Introduction:**

Limb‐shaking transient ischemic attack (LS‐TIA) is a clinical disease with severe carotid stenosis, which is characterized by unilateral rhythmic dance or tremor like involuntary movements of arms and/or legs, but facial muscles are usually unaffected.

**Methods:**

Today, we report a 42‐year‐old man with transient ischemic attack who suffered from right limb shaking and right facial muscle twitching due to the obvious stenosis of left internal carotid artery (ICA). Written informed consent was obtained from participants according to the Declaration of Helsinki, and a local ethic committee approved the study. ICA angioplasty and stent implantation were performed as treatment attempts. A brain protection device was navigated through the lesion and placed at the distal end of the stenosis.

**Result:**

The patient successfully completed the recanalization through stent placement, and the involuntary shaking of limbs and face was improved. During the 3‐month follow‐up, the patient's symptoms disappeared completely and did not attack again.

**Conclusion:**

This case report highlights the importance of accurate diagnosis and treatment, because treatment‐related carotid artery occlusion can not only eliminate the attack, but also reduce the risk of future stroke.

## Graphical abstract

We first report a case of severe left internal carotid artery stenosis leading to limb shaking and facial muscle twitching，and the patient successfully received ICA stent implantation to improve the symptoms.

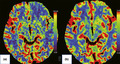

## 1 INTRODUCTION

Limb‐shaking transient ischemic attack (LS‐TIA) is a special and rare type of transient ischemic attack, mainly manifested as involuntary movement of limbs, which is often confused with focal motor epilepsy. It was first reported by Miller Fisher in 1962 and is characterized by brief, jerky, coarse, involuntary movements involving an arm or leg (Fisher, 1962). Most of these clinical symptoms are short‐lived, recurrent, sometimes induced by specific movements or postures. When symptoms occur, patients often describe the decline of coordination ability, inflexibility, and uncontrollability of limbs. Hemodynamic damage of anterior circulation was considered related to this clinical manifestation (Ali, Khan, & Khealani, 2006; Swinnen, Schreurs, Heye, & Lemmens, 2015).

We report a case of severe left internal carotid artery (ICA) stenosis leading to limb shaking and facial muscle twitching, which is the manifestation of brain perfusion damage. And the patient successfully received ICA stent implantation to improve the symptoms.

## 2 CASE REPORT

A 42‐year‐old man presented with transient episodes of clumsiness and shaking of the right arm and leg, accompanied by rhythmic involuntary twitching of the right facial muscles. At times, these episodes would be accompanied by right limb weakness and slurred speech. The episodes would occur at 3–4 Hz, lasting for 1–3 min each in duration, symptoms can disappeared by changes in body position or lying down for a few seconds. The physical symptoms of this patient developed gradually and worsened with the onset of exercise, but not immediately. These symptoms appear at least four times a day and last for more than half a month. He was diagnosed with “focal motor seizures” in other hospitals and “carbamazepine” was given orally in the morning and evening, 200 mg per day, but did not prevent the recurrence of the right limb‐shaking and right facial muscles involuntary movements. The patient had a past history of diabetes and hypertension for 10 years and was on anti‐hypertensive and oral hypoglycemic medications. His medical history shows that his diabetes and high blood pressure have been well controlled, and he is a chronic smoker (one pack a day for the past 10 years). No other neurologic deficits such as parkinsonism or Hyperlipidemia were found. There was no family history of neurologic disorders.

On examination, He is alert and anxious. His blood pressure is 135/74 mmHg, his heart rate is 68 beats/min, and his temperature is 36.8°C. His neurology and general examination showed no pathological changes.

Results of laboratory investigations, including C‐reactive protein levels, glucose, blood cell counts, vitamin B12, serum ammonia levels, liver, and kidney function, urine test, thyroid function, and serum catecholamine were all negative or within the normal range. LDL‐C was 2.25 mmol/L, HbA1c was 6.4%. Further investigations were needed. 24‐hr dynamic electrocardiogram is normal. Repeated interictal electroencephalography (EEG) and Video‐EEG tracing during the course of hospitalization, display the discrete focal theta was found in the left hemisphere without epilepsy. Carbamazepine's attempt to treat epilepsy was stopped when his symptoms did not improve.

**FIGURE 1:**
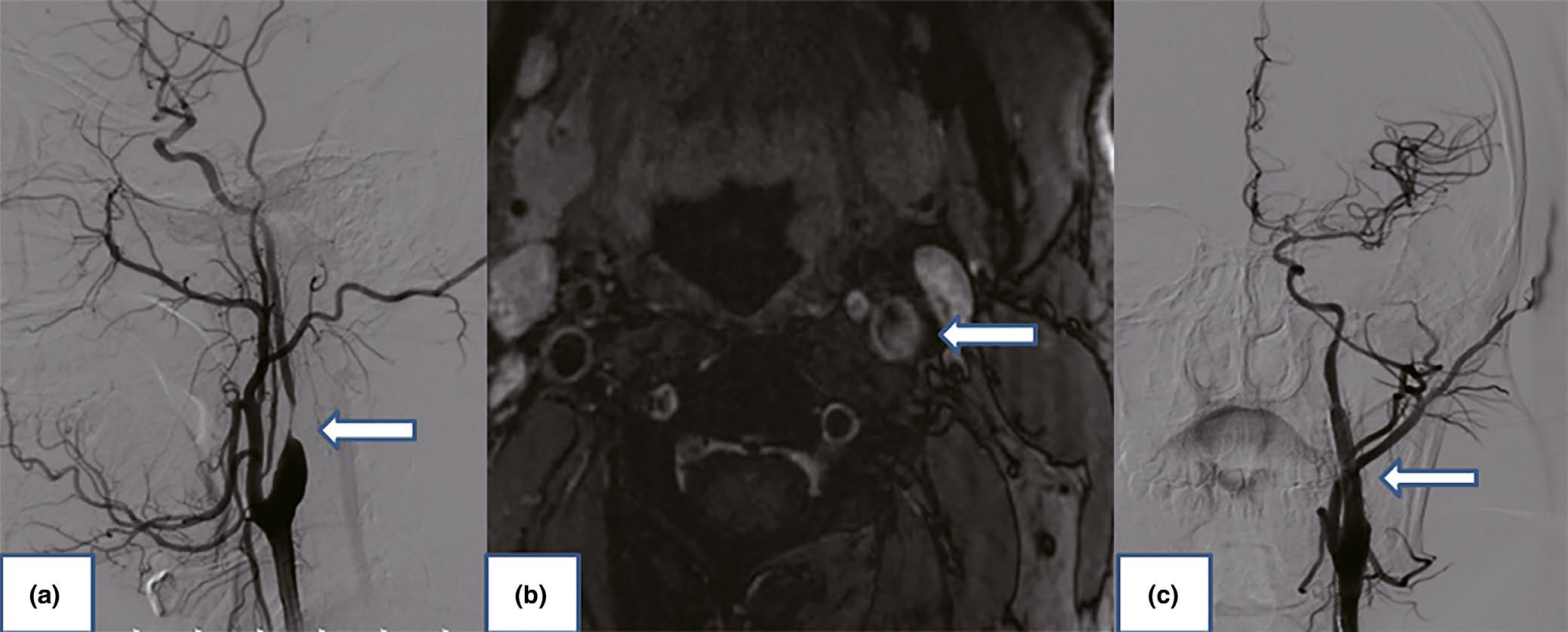
(a) Digital subtraction angiography showing the significant stenosis of the left internal carotid artery (arrow). (b) High‐resolution magnetic resonance imaging showed ruptured fibrous caps, large lipid core, intraplaque hemorrhage, and ulcer (arrow). (c) Completion angiogram after angioplasty and stenting of the left internal carotid artery showing the excellent restoration of flow (arrow)

Color Doppler scan depicted significant stenosis of the left ICA. Then, underwent a four‐vessel cerebral angiography to further clarify his cerebrovascular status, which revealed severe stenosis of his left ICA (90%) (Figure 1a). High‐resolution magnetic resonance imaging showed a large heterogeneous plaque at the beginning of the left internal carotid artery. The ruptured fibrous cap, large lipid nuclei, intraplaque hemorrhage, and ulcers were reviewed in the plaque, indicating the unstable characteristics of the lesion (Figure 1b). MRI of the brain revealed no signs of acute ischemia. The diagnosis of limb‐shaking TIA caused by left hemisphere perfusion impairment was considered. The patient was given fluid replacement therapy, antiplatelet therapy (Aspirin 100 mg/day and Clopidogrel 75 mg/day), and statins (Atorvastatin 20 mg/day), but it showed no significantly improvement of his symptoms. ICA angioplasty and stent implantation were performed as treatment attempts 1 week later. A brain protection device was navigated through the lesion and placed at the distal end of the stenosis. Stent placement and balloon angioplasty were performed before deployment, and excellent restoration flow was restored after operation (Figure 1c).

**FIGURE 2:**
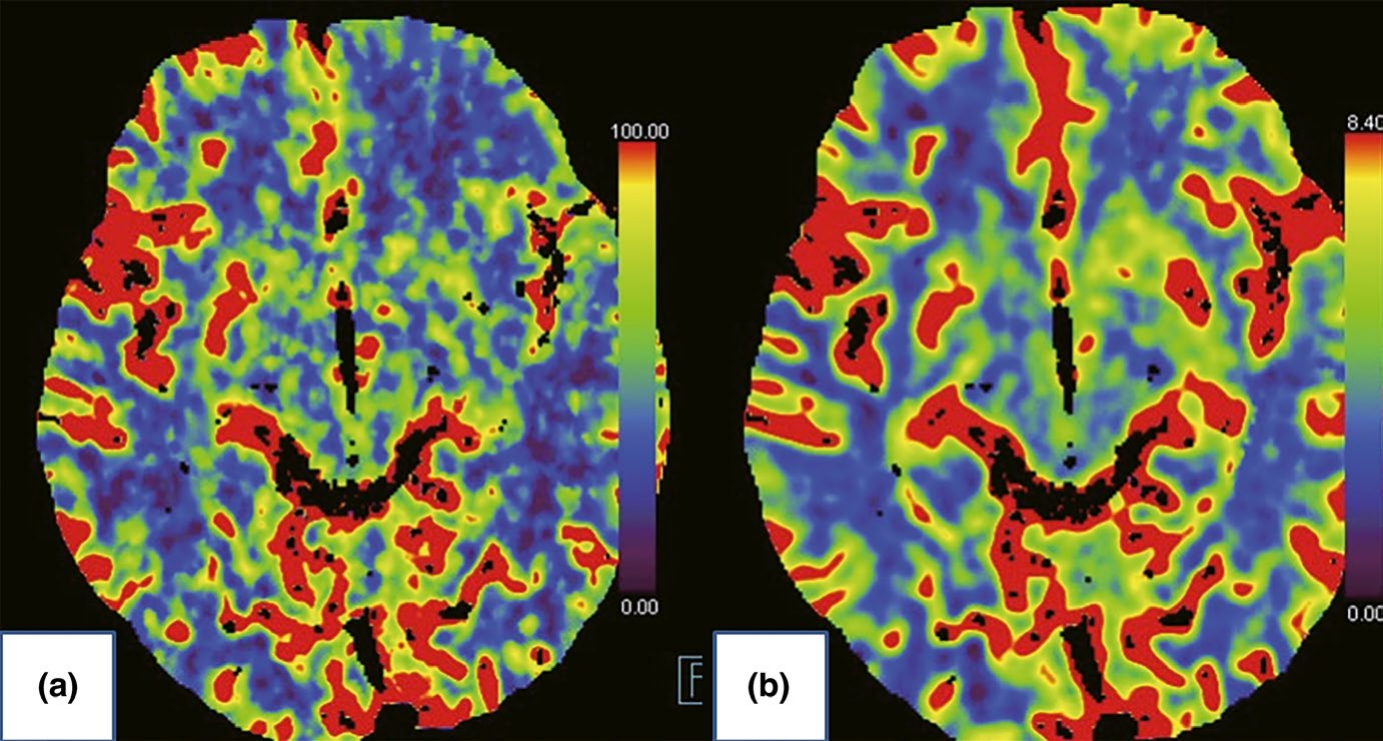
Computed tomography brain perfusion imaging. (a) Preoperative impaired left anterior frontal lobe, the left temporal lobe, and the left basal ganglia blood flow (blue). (b) Postoperative improvement

No neurological dysfunction after operation. He was maintained on antiplatelet therapy and statins after stenting, and no recurrences of his limb‐shaking TIA and facial muscles involuntary twitch. Postoperative recovery was uneventful, and the procedure was successful in completely resolving his symptoms. Color duplex scan showed patency of the ICA with no signs of restenosis and postoperative CT brain perfusion imaging also showed improved left hemispheric cerebral blood flow 3 months later (Figure 2). He received left ICA stent implantation and successfully solved the blood flow in the left hemisphere. His symptoms resolved, and after at least 3 months, he had no more symptoms and no new TIA.

## 3 DISCUSSION

Limb‐shaking TIA characterized by a group of rhythmic or arrhythmic involuntary hyperkinesias affecting the hand, arm, leg, hand‐arm, or hand‐arm‐leg unilaterally. Patients always describe these movements as lack of coordination. The shaking spells occur from single episodes to several times a day. Each attack last <5 min and comes often aggravated by standing up or exercise. It can best be classified as a vascular paroxysmal dyskinesia and considered to be hemodynamic impairment of the anterior circulation and, but face muscles are always spared (Fisher, 1962; Nedelmann, Kolbe, Angermueller, Franzen, & Gizewski, 2011; Siniscalchi, Gallelli, Malferrari, & De Sarro, 2012). Here, we first report a unique case of right limb‐shaking TIA with right facial muscles involuntary twitch, which was induced by significant stenosis of the left ICA, and successfully treated with left internal carotid artery stenting.

Patients with carotid atherosclerosis usually present with a carotid bruit, ischemic symptoms or stroke. TIA may be due to either low flow or embolization (Javaid & Alfishawy, 2016). The symptoms of TIA are related to the vascular territories involved, they are brief, repetitive, stereotyped spells (Fisher, 2002). Limb‐shaking TIA, as a rare form of TIA, is often confused with focal motor seizures. There are important clinical clues which help in differentiating these episodes from the seizure: lack of aura; absence of incontinence; absence of loss of consciousness; absence of tongue bite or a Jacksonian march. Video‐EEG is always normal, and anticonvulsants are ineffective (Baquis, Pessin, & Scott, 1985). These distinctions are crucial, as this form of TIA is usually a sign of severe carotid stenosis and patients are at high risk of future stroke (Baumgartner & Baumgartner, 1998; Das & Baheti, 2013).

Color duplex scan, as less invasive technique, should be first performed to clarify carotid atherosclerosis or stenosis when EEG recording is normal or antiepileptic therapy was ineffective. We used perfusion CT, which demonstrated the hypoperfusion caused by carotid stenosis affecting the left anterior frontal lobe, the left temporal lobe, and the left basal ganglia. This severe hypoperfusion leads to contralateral motor dysfunction. Considering that his symptoms had not resolved and the temporary occlusion required for carotid endarterectomy may exacerbate the cerebral ischemia, carotid stent implantation was performed as treatment attempts. A protection device for the prevention of embolic events during the procedure was used, as the atherosclerotic lesion of the carotid bifurcation was a significant source of emboli. Fortunately, his movement disorder was resolved following carotid stenting and with no neurological dysfunction, and the cerebral perfusion deficit had improved at 3‐month follow‐up later. It was demonstrated that the right limb‐shaking and facial muscles arrhythmic involuntary twitch in this patient were associated with hypoperfusion induced by severely stenosis of the left ICA.

In conclusion, the case of atherosclerotic ICA stenosis that presented with limb‐shaking TIA and facial muscles involuntary twitch was successfully treatment applied to endovascular stenting. Importantly, the timely treatment of the associated carotid artery occlusion may not only abolish the attacks in the patients but also reduce their risk of future stroke.

## CONFLICT OF INTEREST

The authors declare no conflict of interest.

## AUTHOR CONTRIBUTION

We all cared for the patient and contributed to writing of the report. Dong QI and Xiao‐dong Chen were responsible for stent placement and balloon angioplasty. Yuan‐yuan Han was responsible for the intellectual content of the report. Chun‐jie Song was responsible for the data and the figures.

## Data Availability

Data sharing is not applicable to this article as no new data were created or analyzed in this study.
